# Parental knowledge–attitude–practice profiles in childhood myopia: a comprehensive evaluation integrated with clinical findings

**DOI:** 10.1007/s00417-026-07272-7

**Published:** 2026-05-12

**Authors:** Osman Parca, Tunahan Akyol, Alperen Bahar, Emine Seker-Un, Zehra Nur Abadanoglu

**Affiliations:** 1 Department of Ophthalmology, Pamukkale University Faculty of Medicine, Denizli, Türkiye; 2 Department of Ophthalmology, Buldan State Hospital, Denizli, Türkiye; 3 Department of Ophthalmology, SBU Dışkapı Yıldırım Beyazıt Training and Research Hospital, Ankara, Türkiye; 4 Department of Ophthalmology, Göz Akademi Hospital, Denizli, Türkiye; 5 Pamukkale University Faculty of Medicine, Denizli, Türkiye

**Keywords:** Childhood myopia, Parental knowledge, Attitudes, Practices, Myopia progression, KAP model

## Abstract

**Purpose:**

Childhood myopia is increasing globally, yet limited evidence exists on how parental knowledge, attitudes, and practices (KAP) relate to children’s myopia status. This study aimed to evaluate parental KAP regarding childhood myopia and examine their associations with refractive outcomes.

**Methods:**

This cross-sectional study included parents of myopic children aged 6–18 years presenting to an ophthalmology clinic between July and November 2025. A structured KAP questionnaire assessed parental knowledge of risk factors and control methods, attitudes toward myopia severity and management, and daily preventive practices. KAP scores were analyzed as continuous variables. Annual myopia progression was categorized according to refractive change (< − 0.50 D, 0.50–1.00 D, > 1.00 D).

**Results:**

A total of 303 parents were enrolled. The mean knowledge, attitude, and practice scores were 4.68 ± 2.44, 8.12 ± 3.37, and 3.59 ± 1.52, respectively. Higher educational level, urban residence, and a family history of myopia were consistently associated with higher KAP scores. Parental KAP also varied by the child’s myopia severity: parents of children with high myopia demonstrated markedly higher knowledge and attitude scores than those of children with mild or moderate myopia. Correlation analysis showed a moderate positive association between knowledge and attitude scores (*r* = 0.360, *p* < 0.001). Annual myopia progression was negatively correlated with knowledge (*r* = − 0.263, *p* < 0.001), attitude (*r* = − 0.127, *p* = 0.027), and practice scores (*r* = − 0.123, *p* = 0.033), indicating that children of parents with higher KAP scores tended to show less refractive progression.

**Conclusions:**

Parental KAP profiles were associated with clinically relevant patterns of refractive progression in childhood myopia. Despite generally positive attitudes, substantial gaps persisted in parental knowledge and daily implementation of protective practices. Strengthening parental education and improving access to evidence-based interventions may support better behavioral engagement in myopia management.

**Supplementary Information:**

The online version contains supplementary material available at 10.1007/s00417-026-07272-7.

## Key messages

*****What is known***:**

- Parental knowledge, attitudes, and daily practices are key determinants of children’s lifestyle behaviors and are believed to influence modifiable risk factors for childhood myopia, such as outdoor time, near work, and screen exposure.

*****What is new***:**

- Provides one of the first multidimensional assessments linking parental KAP profiles with children’s reported myopia progression, showing that parental behavioral patterns may meaningfully reflect the clinical course.
- Reveals substantial gaps in parents’ understanding of evidence-based myopia-control strategies—including atropine, orthokeratology, and specialized lenses—despite high levels of concern, highlighting a pronounced knowledge–practice gap.
- Demonstrates that higher parental knowledge is positively aligned with both attitudes and protective behaviors, emphasizing the potential impact of integrated parental education and community-based interventions in slowing childhood myopia progression.

**Supplementary Information:**

The online version contains supplementary material available at 10.1007/s00417-026-07272-7.

## Introduction

Childhood myopia has been increasing rapidly worldwide, becoming a significant public health issue. Projections suggest that by 2050, almost half the world’s population could be affected [1]. This rise reflects not only a greater burden of refractive error but also a substantially increased risk of vision-threatening complications, including retinal detachment, myopic maculopathy, glaucoma, and cataract [2, 3]. As a result, managing myopia progression in early childhood is now a key focus in ophthalmology.

Although genetics influences myopia development, lifestyle factors are crucial for progression. Insufficient outdoor time, prolonged near work, and increased digital screen use are recognized risk factors for both onset and progression [4–7]. Evidence-based interventions—incl

uding low-dose atropine, orthokeratology, specialized spectacle lenses, and multifocal contact lenses—can significantly slow myopia progression. These results underline the importance of both lifestyle changes and validated therapies in pediatric myopia management [8–11].

Growing evidence from knowledge, attitudes, and practices (KAP) studies across different populations shows that parents generally perceive myopia as a significant health issue, yet awareness of scientifically proven myopia-control strategies remains limited [12–14]. While some research outlines parental knowledge, attitudes, and practices, fully analytical KAP models that link these elements are lacking [13]. Furthermore, parental knowledge and attitudes often do not reliably lead to corresponding changes in children’s behavior, revealing a gap between knowledge and practice [14]. In addition, only limited data have examined parental awareness or behavioral factors in relation to objective clinical indicators of myopia progression, and multidimensional parental KAP analyses linked to clinical progression data remain scarce [14, 15].

Within this context, adopting a multidimensional KAP framework offers a valuable opportunity to understand how parental behaviors may shape childhood myopia progression. Evidence from Turkish clinical populations also remains limited, although sociocultural context, health literacy, and access to myopia-control options may influence how parental awareness is translated into practice. Therefore, this study aims to comprehensively evaluate parents’ awareness of childhood myopia, their knowledge of available control methods, their attitudes toward myopia-management strategies, their daily preventive practices, and how these factors relate to their children’s reported myopia progression.

## Methods

This study was a descriptive, cross-sectional investigation designed to evaluate parental KAP regarding childhood myopia. Parents of myopic children aged 6–18 years who presented to the ophthalmology outpatient clinic between July and November 2025 were recruited using a consecutive sampling approach. Inclusion criteria were parental literacy, willingness to complete the questionnaire, and a confirmed diagnosis of myopia in the child. Exclusion criteria included any ocular pathology or cognitive impairment that could interfere with completing the questionnaire. Written informed consent was obtained from all participants, and the study was approved by the Pamukkale University Non-Interventional Clinical Research Ethics Committee in accordance with the Declaration of Helsinki (Approval No: E-60116787-020-728571, 29.07.2025).

The questionnaire was developed based on previously published parental myopia surveys and standard KAP survey frameworks [13, 16, 17]. Its content and clarity were reviewed by three ophthalmologists experienced in pediatric myopia management. No pilot study, internal consistency analysis, or formal psychometric validation was performed, because the primary aim of the study was to obtain a cross-sectional situational assessment rather than to validate a new measurement instrument. The survey included items on demographic characteristics, the child’s myopia profile, parental knowledge of myopia and evidence-based control methods, attitudes toward myopia severity and management strategies, and the child’s daily lifestyle practices. Parents completed the questionnaire face-to-face using a paper format, typically within 8–10 min.

Separate scoring methods were used for knowledge, attitude, and practice. For knowledge, correct answers were scored as ‘1’ and incorrect answers as ‘0’. In the attitude domain, higher scores reflected greater awareness of the seriousness of childhood myopia and more positive views toward evidence-based control strategies. In the practice domain, higher scores indicated more protective behaviors, such as greater outdoor exposure, screen-time regulation, healthier near-work habits, and regular eye examinations. In accordance with standard KAP methodology, knowledge, attitude, and practice were treated as conceptually distinct domains; therefore, each domain was analyzed separately as a continuous variable rather than combined into a single composite score or converted into categorical groups. This approach was adopted to preserve domain-specific differences and to allow a more precise evaluation of parental profiles across the three KAP dimensions. Information about myopia progression was obtained by comparing clinical records and the most recent eyeglass prescriptions. Annual refractive changes were classified as < 0.50 D, 0.50–1.00 D, or > 1.00 D.

Because the questionnaire was administered face-to-face, no item-level missing questionnaire data were present among the included participants. However, annual myopia progression could not be classified in 18 children because sufficient prescription or clinical follow-up data from the preceding year were unavailable; these cases were retained in the descriptive analyses but excluded from correlation analyses involving myopia progression.

A sample size calculation was performed using G*Power 3.1. As no prior local dataset was available to support an effect-size-based calculation for a multidimensional parental KAP analysis, a prevalence-based approach was applied. Based on a recent European systematic review and meta-analysis reporting a childhood myopia prevalence of 23.5% (95% CI: 18.5–29.3), and assuming a 95% confidence level, α = 0.05, and 80% power, the minimum required sample size was estimated as 270 [18]. The final sample comprised 303 participants, exceeding this threshold.

This study was designed as a descriptive cross-sectional analysis and did not include a multivariable adjustment model. Instead, the available background variables, including parental education level, residence type, and number of myopic parents, were examined in subgroup analyses. Data were analyzed using SPSS version 26. Continuous variables were presented as mean ± standard deviation or median, and categorical variables as frequencies and percentages. The distribution of KAP scores was assessed using the Kolmogorov–Smirnov test. Group differences were evaluated using the Mann–Whitney U test or Kruskal–Wallis test, as appropriate. Correlations among continuous KAP domain scores were assessed using Pearson’s correlation analysis, whereas associations between KAP scores and ordinal myopia progression categories were evaluated using Spearman’s correlation analysis. A p-value < 0.05 was considered statistically significant.

## Results

A total of 303 parents were included in the study. The mean age of the children was 13.67 ± 3.7 years, and 54.5% were female. The mean age at myopia onset was 9.79 ± 2.9 years. Based on spherical equivalent, 63.5% of the children had mild myopia, 30.2% had moderate myopia, and 6.3% had high myopia. Annual myopia progression was < 0.50 D in 51.2% of participants, 0.50–1.00 D in 34.3%, and > 1.00 D in 8.6% (Table 1).

**Table 1:** Demographic and clinical characteristics of study participants

Characteristic		*n* (%)/Mean ± SD
Child’s Age (years)		13.67 ± 3.7
Age at onset of myopia (years)		9.79 ± 2.9
Child’s sex	Male	138 (45.5)
	Female	165 (54.5)
Residence type	Rural	52 (17.2)
	Urban	251 (82.8)
Parental education level	Middle school	92 (30.3)
	High school	93 (30.7)
	University	118 (38.9)
Parental myopia	None	133 (43.9)
	One	127 (41.9)
	Two	43 (14.2)
Spherical equivalent refractive error (D)	–−0.50 to − 3.00 D	191 (63.5)
	–3.25 to −5.75 D	91 (30.2)
	≤ − 6.00 D	19 (6.3)
Annual myopic progression (D/year)	< − 0.50 D	155 (51.2)
	0.50–1.00 D	104 (34.3)
	> 1.00 D	26 (8.6)
	Unknown	18 (5.9)

*SD *standard deviation*, D *diopters

The mean parental KAP scores were 4.68 ± 2.44, 8.12 ± 3.37, and 3.59 ± 1.52, respectively. A summary of the item-level response distributions is presented in Table 2, and detailed percentages for each item are provided in Supplementary Table S1.

**Table 2:** Summary distribution of parental responses to knowledge, attitude, and practice items

Survey Item			Yes *n* (%)
Knowledge	Do you know that a child’s lifestyle habits influence the development of myopia?		261 (86,1)
		Highest awareness: Prolonged screen use	245 (80,9)
		Lowest awareness: Insufficient outdoor time	48 (15,8)
Attitude	Which myopia control method(s) would you consider applying?		277 (91,4)
		Most preferred method: Spectacle lenses designed for myopia control	191 (63)
		Least preferred method: Overnight orthokeratology lenses	23 (7,6)
Practice	Which vision correction method is your child currently using?		
		Conventional single-vision spectacles	246 (81,2)
		Spectacle lenses designed for myopia control	40 (13,2)
		Soft contact lenses	17 (5,6)

Significant differences were observed in knowledge, attitude, and practice scores across sociodemographic variables (Table 3). Higher educational attainment was associated with higher KAP scores. Parents residing in urban areas had higher knowledge scores than those living in rural regions. A positive association was also identified between a family history of myopia and both knowledge and attitude scores. No significant differences were found by children’s sex (*p* > 0.05).

**Table 3:** Comparison of knowledge, attitude, and practice scores according to sociodemographic variables

Variable	*n* (%)	Knowledge Score (Mean ± SD)	Attitude Score (Mean ± SD)	Practice Score (Mean ± SD)
Child’s Sex				
Male	138 (45.5)	4.90 ± 2.5	8.48 ± 3.3	3.64 ± 1.5
Female	165 (54.5)	4.50 ± 2.4	7.82 ± 3.3	3.54 ± 1.5
*p-value*		0.468	0.477	0.560
Residence				
Rural	52 (17.2)	3.31 ± 1.8	5.39 ± 3.0	3.14 ± 1.3
Urban	251 (82.8)	4.91 ± 2.4	8.59 ± 3.1	3.67 ± 1.5
*p-value*		0.026	0.231	0.147
Parental education level				
Middle school	92 (30.3)	4.12 ± 2.3	6.43 ± 3.5	3.49 ± 1.4
High school	93 (30.7)	4.00 ± 2.1	8.49 ± 3.1	3.34 ± 1.4
University	118 (38.9)	5.68 ± 2.3	9.08 ± 2.9	3.87 ± 1.5
*p-value*		< 0.01	< 0.01	0.047
Number of myopic parents				
None	131 (43.2)	4.03 ± 2.4	7.02 ± 3.4	3.56 ± 1.5
One	127 (41.9)	5.11 ± 2.4	8.91 ± 3.0	3.62 ± 1.4
Two	43 (14.2)	5.49 ± 2.1	9.07 ± 3.2	3.60 ± 1.6
*p-value*		< 0.01	< 0.01	0.774

Data are presented as mean ± standard deviation (SD) or percentage (%)

p-values were calculated using the Mann–Whitney U test for two-group comparisons and the Kruskal–Wallis test for comparisons involving three groups

Significant differences in knowledge, attitude, and practice scores were observed across myopia severity groups (Kruskal–Wallis: knowledge, H = 16.85, *p* < 0.001; attitude, H = 19.64, *p* < 0.001; practice, H = 12.62, *p* < 0.01). Post hoc Dunn–Bonferroni analyses revealed that the high myopia group had significantly higher knowledge and attitude scores than both the mild and moderate myopia groups (knowledge: mild–high *p* < 0.0001, moderate–high *p* < 0.01; attitude: mild–high *p* < 0.0001, moderate–high *p* < 0.0001). In terms of practice scores, the moderate myopia group had lower scores than both the mild and high myopia groups (mild–moderate *p* < 0.01; moderate–high *p* < 0.05). No significant difference was observed between the mild and high myopia groups for practice scores.

**Fig. 1:**
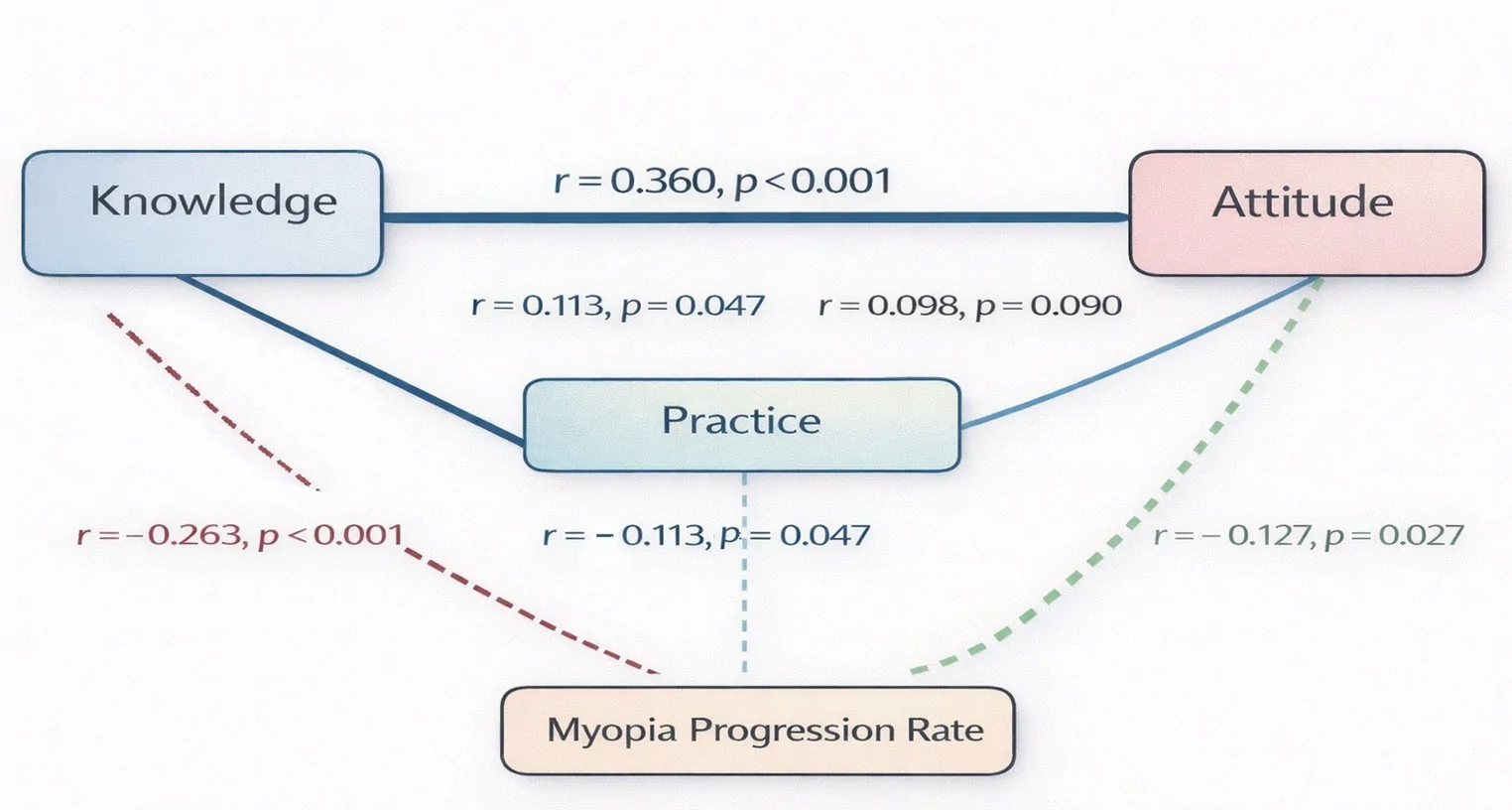
Correlation diagram summarizing the relationships among parental knowledge, attitude, and practice scores and annual myopia progression. Solid lines indicate positive correlations, dashed lines indicate negative correlations, and dotted lines indicate non-significant correlations. Line thickness is proportional to correlation strength, with correlation coefficients (r) and p-values shown for each association

A summary of the correlation analyses is presented in Fig. 1. Knowledge and attitude scores showed a moderate positive correlation (*r* = 0.360, *p* < 0.001). The correlations of knowledge with practice (*r* = 0.113, *p* = 0.047) and of attitude with practice (*r* = 0.098, *p* = 0.090) were weaker. Myopia progression demonstrated negative correlations with all KAP components, including knowledge (*r* = − 0.263, *p* < 0.001), attitude (*r* = − 0.127, *p* = 0.027), and practice scores (*r* = − 0.123, *p* = 0.033), indicating that higher knowledge, attitude, and practice scores were associated with slower reported progression.

## Discussion

Our study provides important insight into how parental behaviors—an increasingly critical yet insufficiently understood aspect of childhood myopia management—relate not only to knowledge but also to actual practices and clinical outcomes. Our findings suggest that, in addition to parents’ limited fundamental understanding of myopia, they experience notable difficulties in implementing protective behaviors, and these challenges appear to overlap with groups reporting more pronounced myopia progression. This relationship indicates that evaluating questionnaire-based KAP components alongside clinical progression data may offer a critical framework for identifying behavioral and structural barriers in myopia control.

One of the most clinically relevant and potentially distinctive findings of this study is the observed association between parental KAP profiles and myopia progression. The negative correlations between annual refractive change and all KAP components suggest that children of parents with higher knowledge, more favorable attitudes, and better practices may experience slower progression. In addition, the inverse associations between age at myopia onset and KAP scores imply that earlier recognition of myopia and its potential consequences may increase the likelihood that families adopt protective behaviors and consider myopia control strategies. Although these relationships should be interpreted cautiously in view of the cross-sectional design, they suggest that parental KAP profiles may reflect more than theoretical awareness alone and may be meaningfully linked to clinically relevant progression patterns. From a practical perspective, this finding supports the potential value of KAP-based evaluation in identifying families who may benefit from earlier education, closer counseling, and more targeted myopia control interventions.

Although parents showed relatively good awareness of certain risk factors—particularly screen time—important knowledge gaps were identified in areas such as outdoor activity, sleep, and evidence-based myopia control strategies. This pattern is consistent with previous KAP studies, which have identified awareness of myopia control methods as one of the weakest domains [13, 17, 19]. Similarly, earlier studies have reported limited parental familiarity with clinically effective interventions such as atropine, orthokeratology, and specialized optical treatments [20]. These findings suggest that although families may better understand the behavioral aspects of myopia, they face substantial challenges accessing detailed, scientifically grounded information on intervention strategies.

Another notable finding of this study is the high level of parental concern regarding childhood myopia and the generally positive attitudes toward protective interventions, consistent with previous studies reporting that parents tend to perceive myopia as a serious condition [19]. However, this favorable attitude was not fully translated into daily behavior. In our cohort, many children had limited outdoor exposure, suboptimal sleep patterns, and very low use of evidence-based interventions such as low-dose atropine, orthokeratology, and specialized spectacle lenses. A similar attitude–practice gap has been reported across diverse populations. For example, a recent KAP study in Asia showed that, despite high parental motivation to prevent and control myopia, the adoption of practical behaviors remained limited [21]. Likewise, studies evaluating optical interventions have shown that, although parents often view orthokeratology and specially designed spectacle lenses positively, actual utilization remains low because of concerns related to cost, safety, and insufficient information [14]. Together with previous reports showing that implementation is constrained by uncertainty and accessibility barriers rather than knowledge alone [12, 21, 22], our findings suggest that positive attitudes alone are insufficient to produce behavioral change and that parental decision-making is also shaped by structural and practical barriers.

The positive correlations among knowledge, attitude, and practice scores suggest that these domains are closely interrelated. In particular, the association of knowledge with both attitude and practice indicates that parental knowledge may play a central role in shaping both intention and behavior. Despite the overall low practice scores, this pattern supports the view that improving parental knowledge may promote more favorable attitudes and facilitate behavioral change, underscoring the practical relevance of educational interventions in childhood myopia control.

Subgroup analyses further supported these behavioral patterns. Higher educational attainment was associated with higher KAP scores, underscoring the central role of health literacy in myopia management. Similarly, although parents residing in urban areas demonstrated higher knowledge levels, this did not fully translate into practice, suggesting that knowledge alone may be insufficient to drive behavioral change. In addition, the association between family history of myopia and higher knowledge and attitude scores may reflect greater awareness among parents with prior personal or familial experience. Together, these findings suggest that parental KAP profiles are shaped not only by individual characteristics but also by broader sociocultural and environmental context.

Compared with previous research, one of the important strengths of this study is the integration of parental knowledge, attitudes, and practices with clinical progression data. The relatively limited number of studies evaluating KAP domains in such a multidimensional manner while also relating them to annual refractive change increases the clinical relevance of our findings. Additional strengths include the relatively large sample size, the use of data derived from a real clinical population, and the classification of myopia progression based on objective clinical records. Furthermore, analyzing KAP scores as continuous variables and examining sociodemographic subgroups allowed a more nuanced interpretation of parental behaviors related to childhood myopia.

Several methodological limitations should be considered when interpreting the results. First, its cross-sectional design precludes causal inference between parental KAP profiles and childhood myopia progression. Second, although the questionnaire underwent expert content review, it was not subjected to formal psychometric validation. Third, socioeconomic status was not directly measured; although parental education level and residence type were evaluated as background variables, residual confounding related to unmeasured socioeconomic factors cannot be excluded. Furthermore, the single-center design and the specific sociodemographic profile of the study population may limit the generalizability of the findings.

Taken together, our findings suggest that parental knowledge, attitudes, and practices regarding childhood myopia may reflect not only behavioral tendencies but also clinically relevant patterns of progression. The results also indicate that patterns commonly reported in the literature—including limited knowledge, positive but insufficient translation into practice, and a persistent knowledge–practice gap—are likewise observed in a real-world clinical population. At the same time, the positive directional relationships among KAP components suggest that strengthening parental education may help support more sustainable behavioral change in childhood myopia management. Integrating evidence-based clinical approaches with family education, school-based programs, and community-level awareness strategies may offer a practical, accessible pathway to reduce myopia progression in children and improve public health outcomes.

## Supplementary Information

Below is the link to the electronic supplementary material.

Supplementary Material 1 (DOCX 32.0 KB)

## Data Availability

The datasets generated and analyzed during the current study are available from the corresponding author on reasonable request.
