# Supplementary material for: Parental knowledge–attitude–practice profiles in childhood myopia: a comprehensive evaluation integrated with clinical findings
Source: Graefes Arch Clin Exp Ophthalmol. 2026 May 12;264(8):2481–7. doi: 10.1007/s00417-026-07272-7 (PMC13490247; doi:10.1007/s00417-026-07272-7)
Supplement: Supplementary file 1 [file 417_2026_7272_MOESM1_ESM.docx]

**Supplementary Table S1** Summary Distribution of Parental Responses to Knowledge, Attitude, and Practice Items

| Survey Items | Yes n (%) |
| --- | --- |
| Knowledge |  |
| Aware that a child’s lifestyle habits influence myopia development | 261 (86.1) |
| Prolonged screen use | 245 (80.9) |
| Insufficient outdoor activity | 48 (15.8) |
| Insufficient sleep duration | 109 (36.0) |
| Poor lighting conditions | 88 (29.0) |
| Aware of potential complications of high myopia | 125 (41.3) |
| Cataract formation | 52 (17.2) |
| Glaucoma development | 12 (4.0) |
| Retinal detachment | 51 (16.8) |
| Aware that myopia control interventions exist | 197 (65.0) |
| Information source: Ophthalmologist | 116 (58.0) |
| Information source: Social media / Internet | 50 (28.0) |
| Information source: Television | 3 (1.5) |
| Awareness of specific myopia control methods | 258 (85.1) |
| Pharmacological treatment (eye drops) | 116 (38.3) |
| Overnight orthokeratology lenses | 38 (12.5) |
| Soft contact lenses for myopia control | 45 (14.9) |
| Spectacle lenses designed for myopia control | 174 (57.4) |
| Attitude |  |
| Concerned about child’s myopia | 280 (92.4) |
| Reason: Ocular health | 248 (81.8) |
| Reason: Dependence on glasses/lenses | 91 (30.0) |
| Reason: Economic burden of glasses/lenses | 8 (2.6) |
| Reason: Aesthetic or personal image | 35 (11.6) |
| Restricts child’s screen time | 218 (72.0) |
| By setting a daily limit | 120 (39.6) |
| By limiting when necessary | 98 (32.3) |
| Would consider applying any myopia control method | 277 (91.4) |
| Pharmacological treatment (eye drops) | 111 (36.6) |
| Overnight orthokeratology lenses | 23 (7.6) |
| Soft contact lenses for myopia control | 48 (15.8) |
| Spectacle lenses designed for myopia control | 191 (63.0) |
| Practice |  |
| Current vision correction method used by child |  |
| Spectacles | 246 (81.2) |
| Spectacle lenses for myopia control | 40 (13.2) |
| Soft contact lenses | 17 (5.6) |
| Frequency of eye examinations |  |
| Every 6 months | 88 (29.0) |
| Annually | 128 (42.2) |
| Every two years | 50 (16.5) |
| Only when visual complaints occur | 29 (9.6) |
| Daily near-work duration |  |
| < 1 hour | 37 (12.2) |
| 1–3 hours | 110 (36.3) |
| > 3 hours | 156 (51.5) |
| Daily outdoor time |  |
| < 1 hour | 83 (27.4) |
| 1–3 hours | 153 (50.5) |
| > 3 hours | 67 (22.1) |
| Daily sleep duration |  |
| < 8 hours | 73 (24.1) |
| 8–9 hours | 134 (44.2) |
| 9–10 hours | 81 (26.7) |
| > 10 hours | 15 (5.0) |
